# MRI Evaluation of Corpus Callosum Malformation and Associated Anomalies: A Retrospective Cross-Sectional Study

**DOI:** 10.7759/cureus.70924

**Published:** 2024-10-06

**Authors:** Saurya Saurya, Bhumika Dhamija, Srishti Sharma, Ravi S Singh, Priyanka KS, Saroj Kumar, Kumar Siddhant, Sumair Haque

**Affiliations:** 1 Department of Radiodiagnosis, All India Institute of Medical Sciences, Patna, Patna, IND; 2 Department of Radiodiagnosis, All India Institute of Medical Sciences, Rishikesh, Rishikesh, IND; 3 Department of Interventional Neuroradiology, All India Institute of Medical Sciences, Patna, Patna, IND

**Keywords:** agenesis of the corpus callosum, commissure, corpus callosum, dandy-walker complex, heterotopia

## Abstract

Objective

This study aims to evaluate the MRI morphology of corpus callosum malformations and the associated anomalies frequently observed in a tertiary care center in northern India.

Methods

We conducted a retrospective cross-sectional study using MRI reports, images, and clinical records from January 2020 to July 2024. A total of 19 patients with corpus callosum agenesis or hypoplasia were identified, with 17 patients included after excluding those with incomplete records. We analyzed MRI findings for agenesis type (complete or partial), commissural abnormalities, midline anomalies, cortical abnormalities, and posterior fossa abnormalities. Statistical analysis was performed using chi-square tests to compare associations between complete and partial agenesis.

Results

Of the 17 patients, 52.9% had complete agenesis and 47.1% had partial agenesis. Complete agenesis was associated with higher rates of commissural involvement (44.4% vs. 12.5%), midline anomalies (22.2% vs. 0%), and Probst bundle formation (88.9% vs. 37.5%). Ventricular distortion was more common in complete agenesis (88.9% vs. 40%), and cortical malformations were also more prevalent (44.4% vs. 12.5%). Other anomalies included holoprosencephaly, Dandy-Walker malformation, and hypoxic-ischemic encephalopathy changes.

Conclusion

Complete agenesis of the corpus callosum is significantly associated with other commissural abnormalities, midline cysts, Probst bundle formation, ventricular distortions, and cortical malformations compared to partial agenesis. This study highlights the varied imaging presentations of corpus callosum malformations and their associated anomalies.

## Introduction

The corpus callosum is the largest commissural fiber of the brain, connecting the two cerebral hemispheres. It is divided into four divisions, from antero-inferior to posterior: rostrum, genus, body, and splenium [1].

The formation of corpus callosum occurs from 10 to 17 weeks of gestation in the anterior to posterior direction, with the genus being formed first and the splenium and rostrum being formed last. The development of two other commissural fibers, i.e., the anterior commissure and hippocampal commissures, is closely associated with the development of the corpus callosum [2].

Abnormality in the formation of the corpus callosum can result in partial or complete agenesis of the corpus callosum. In complete agenesis, the entire corpus callosum is absent, whereas in partial agenesis, the later-formed divisions of the corpus callosum are absent. In tricommissural agenesis, the corpus callosum, anterior, and hippocampal commissures are absent [3].

Corpus callosum agenesis is frequently associated with other anomalies: midline abnormalities like interhemispheric cysts or lipoma and absent septum pellucidum, posterior fossa abnormalities like Dandy-Walker spectrum malformation, and disorders of cortical development and neuronal migration [4].

Agenesis of the corpus callosum is also associated with various genetic syndromes like Apert syndrome, basal cell nevus syndrome, Joubert syndrome, Meckel-Gruber syndrome, septo-optic dysplasia, and Aicardi syndrome [5].

In this study, we evaluate the MRI morphology of corpus callosum malformation and anomalies frequently associated with it, as presented in a tertiary care center in Northern India.

## Materials and methods

Study design

This study was designed as a retrospective cross-sectional study conducted at a tertiary care hospital in northern India. The primary objective was to evaluate the MRI morphology of corpus callosum malformations and the frequency of associated anomalies in patients presenting to the radiology department in a tertiary care hospital between January 2020 and July 2024.

Data collection

Data were collected by performing a comprehensive search in the institutional report archive using specific keywords: “Agenesis Corpus Callosum,” “Hypogenesis Corpus Callosum,” and “Dysgenesis Corpus Callosum.” A total of 19 reports matching these keywords were identified. The clinical record (CR) numbers of these patients were extracted, and detailed clinical information was obtained from the hospital information system (HIS). The MRI images of these patients were retrieved from the institution's picture archiving and communication system (PACS). After retrieval, two patients were excluded due to incomplete clinical records and imaging data. Thus, 17 patients were included in the final analysis.

Inclusion criteria

All cases with confirmed MRI diagnoses of agenesis or hypogenesis of the corpus callosum, accompanied by comprehensive clinical details and imaging records from January 2020 to July 2024, were included in the study.

Exclusion criteria

Patients were excluded if they had incomplete clinical records or missing MRIs that did not allow for a comprehensive evaluation of the corpus callosum malformation and associated anomalies. Cases with thinned-out corpus callosum secondary to hypoxic-ischemic encephalopathy (HIE) changes were also not included in the study.

Statistical analysis

Data were tabulated in Microsoft Excel (Microsoft Corporation, Redmond, WA) under the following categories: age, type of agenesis (partial or complete), commissural abnormalities, midline anomalies, cortical abnormalities, and posterior fossa abnormalities. Descriptive statistics were used to summarize the data. Chi-square tests were conducted to compare the prevalence of associated anomalies between the partial and complete agenesis groups. A p-value of less than 0.05 was considered statistically significant.

## Results

The study included 17 patients, of which nine (52.9%) exhibited complete agenesis of the corpus callosum, while eight (47.1%) showed partial agenesis. Patients with complete agenesis were diagnosed earlier and demonstrated more extensive neurological involvement in imaging than those with partial agenesis (Table 1).

**Table 1:** Table of anomalies and their frequency.

Anomaly	Complete agenesis (n = 9)	Partial agenesis (n = 8)
Commissural involvement	4 (44.4%)	1 (12.5%)
Midline anomalies (cysts/lipoma)	2 cysts (22.2%), 1 lipoma (11%)	0 (0%)
Ventricular distortion	8 (88.9%)	3 (40%)
Probst bundle	8 (88.9%)	3 (37.5%)
Cortical malformations	4 (44.4%)	1 (12.5%)
Other anomalies	5 (55.6%)	3 (37.5%)

Commissural involvement

**Figure 1:**
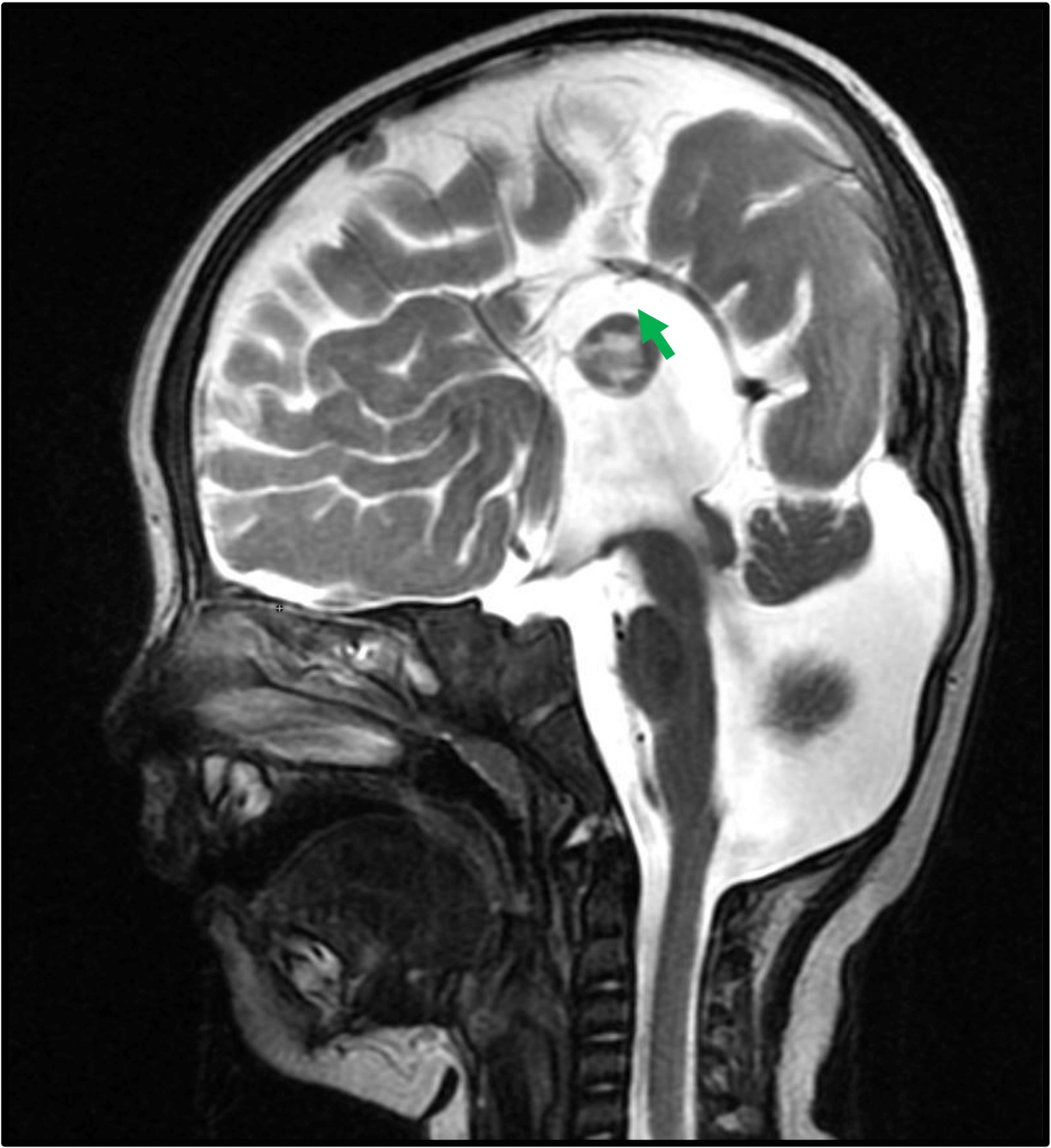
Tricommissural agenesis. Sagittal T2-weighted imaging shows the absence of corpus callosum and anterior and posterior commissure. Associated inferior vermian hypoplasia and enlarged posterior fossa were noted, suggesting Dandy-Walker spectrum malformation.

Commissural involvement (Figure 1) was identified in 44.4% of the patients with complete agenesis (four out of nine patients). In contrast, only 12.5% of the partial agenesis cases (one out of eight patients) exhibited commissural abnormalities. The data showed a statistically significant higher rate of commissural involvement in complete agenesis compared to partial agenesis (p < 0.05).

Midline anomalies: cysts and lipoma

**Figure 2:**
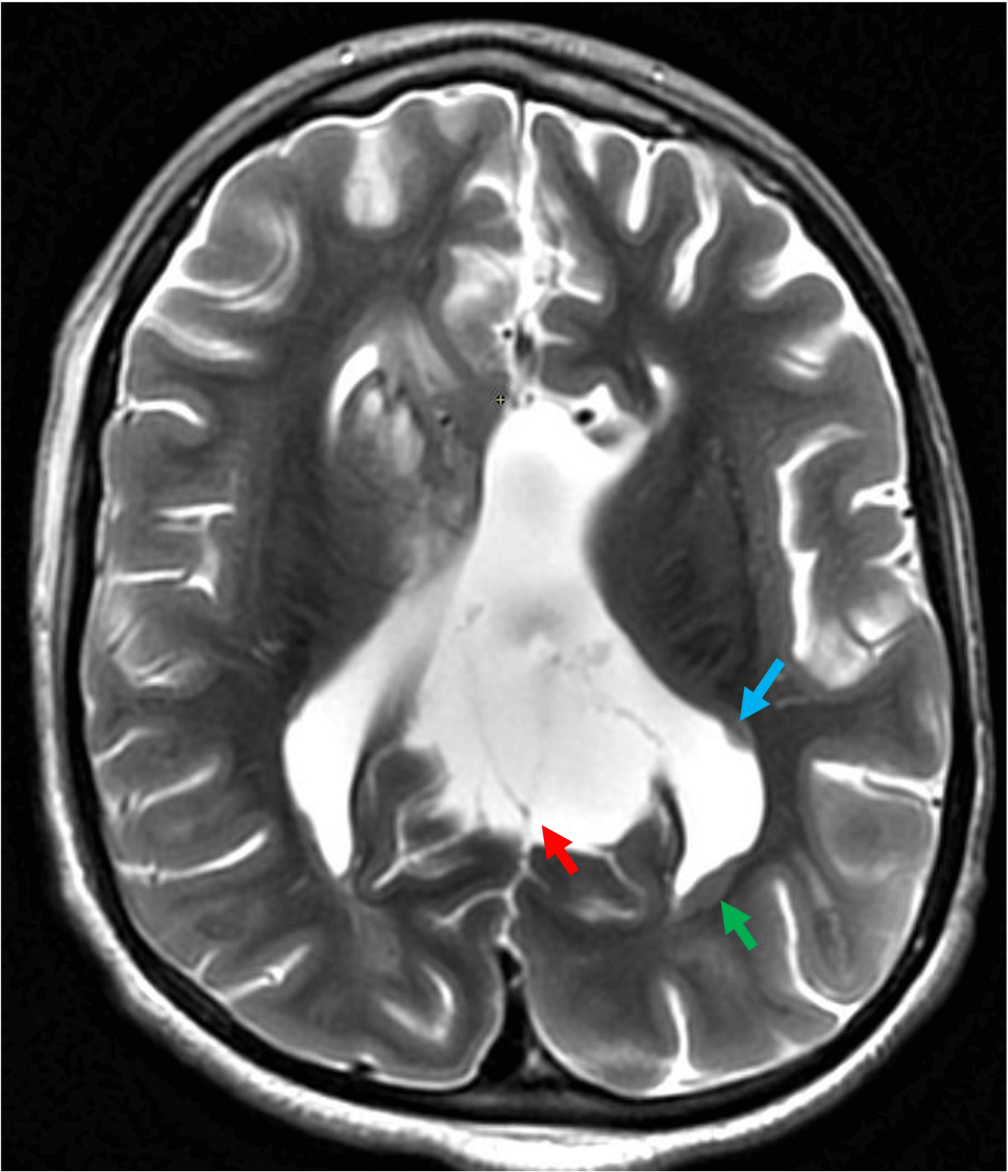
Corpus callosum agenesis with interhemispheric cyst. Axial T2-weighted imaging showing interhemispheric cyst (red arrow), associated with parallelly oriented ventricles (green arrow). Focal periventricular gray matter heterotopia (blue arrow) was noted.

**Figure 3:**
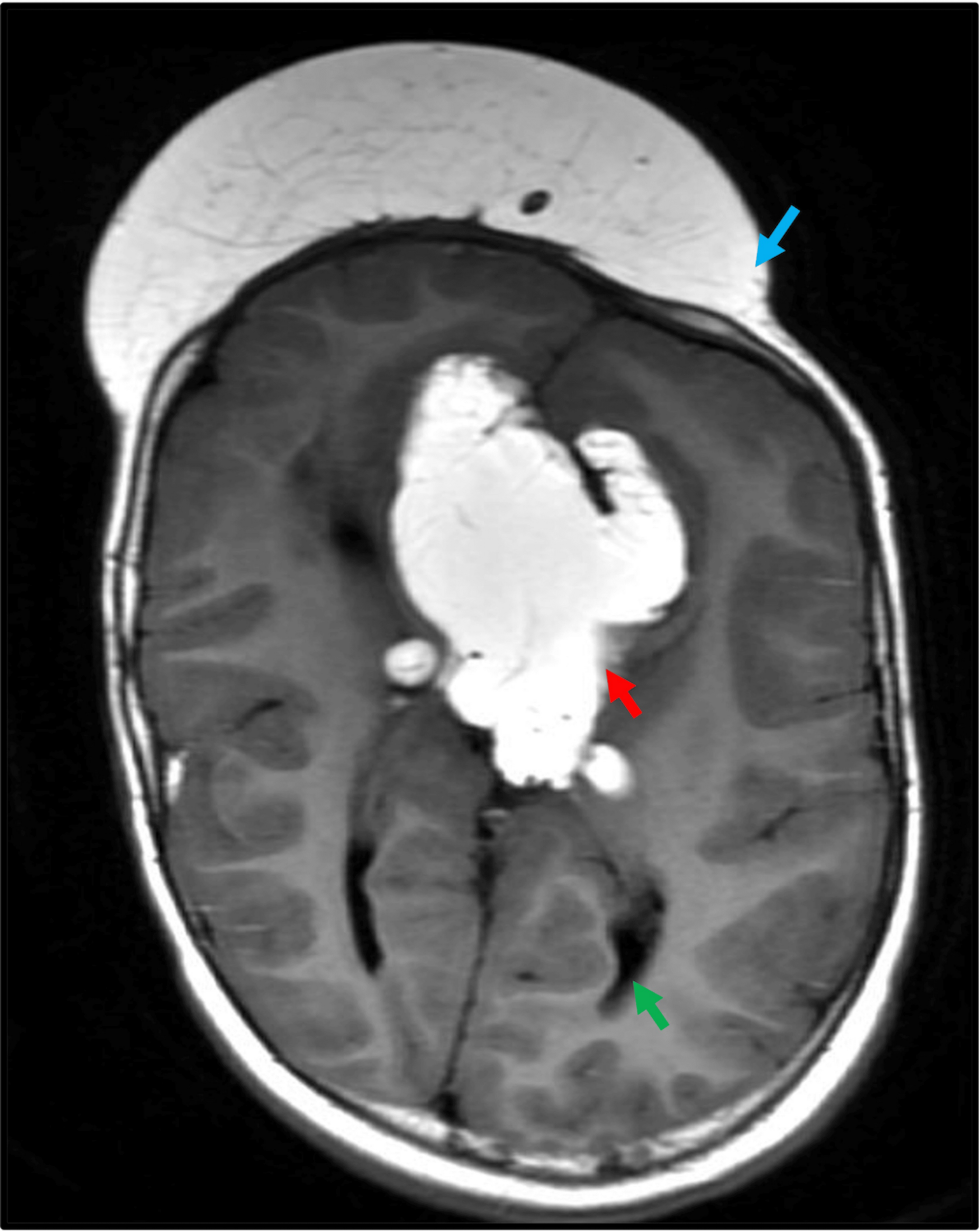
Corpus callosum agenesis with interhemispheric lipoma. Axial T1-weighted imaging showing lobulated T1 hyperintense interhemispheric lesion (red arrow). Note the parallel arrangement of lateral ventricles (green arrow). Note the subcutaneous frontal lipoma (blue arrow).

Midline anomalies were relatively rare in this cohort. Among patients with complete agenesis, interhemispheric cysts (Figure 2) were observed in 22.2% (two out of nine patients), and one patient presented with a lipoma (Figure 3). No midline anomalies were found in patients with partial agenesis. There was a statistically significant association between complete agenesis and the presence of midline anomalies (p < 0.05).

Ventricular distortion

**Figure 4:**
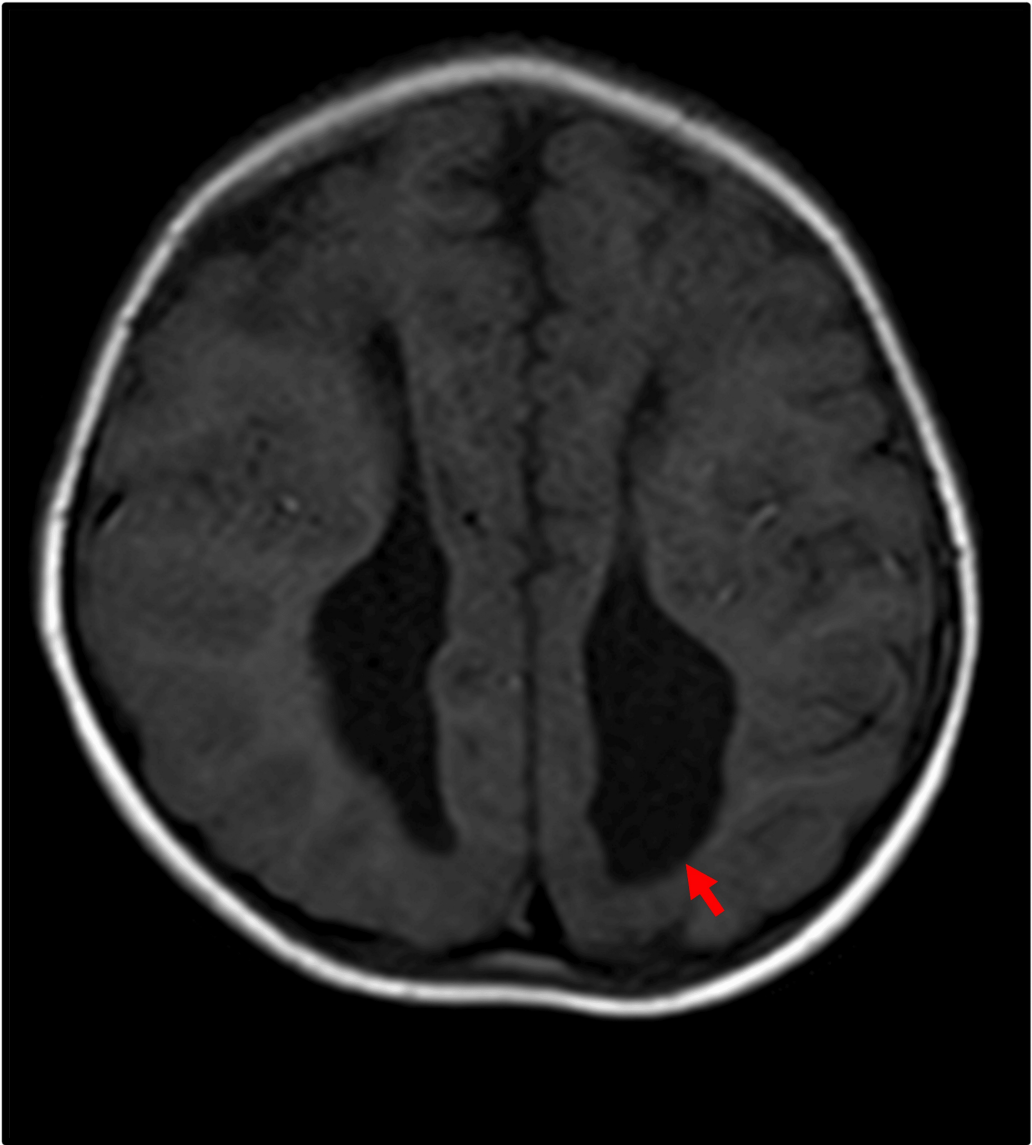
Agenesis of the corpus callosum with colpocephaly. Axial T1-weighted imaging showing the parallel orientation of the lateral ventricles with dilated trigones and occipital horns (red arrow).

Ventricular distortion, primarily colpocephaly (Figure 4), was a common finding. It was present in 88.9% (eight out of nine) of the patients with complete agenesis, while only 40% (three out of eight) of the patients with partial agenesis exhibited ventricular distortion. This difference was statistically significant (p < 0.05). Additionally, other ventricular abnormalities, such as ventricular asymmetry and dysmorphic frontal horns, were noted in one patient each.

Probst bundle identification

**Figure 5:**
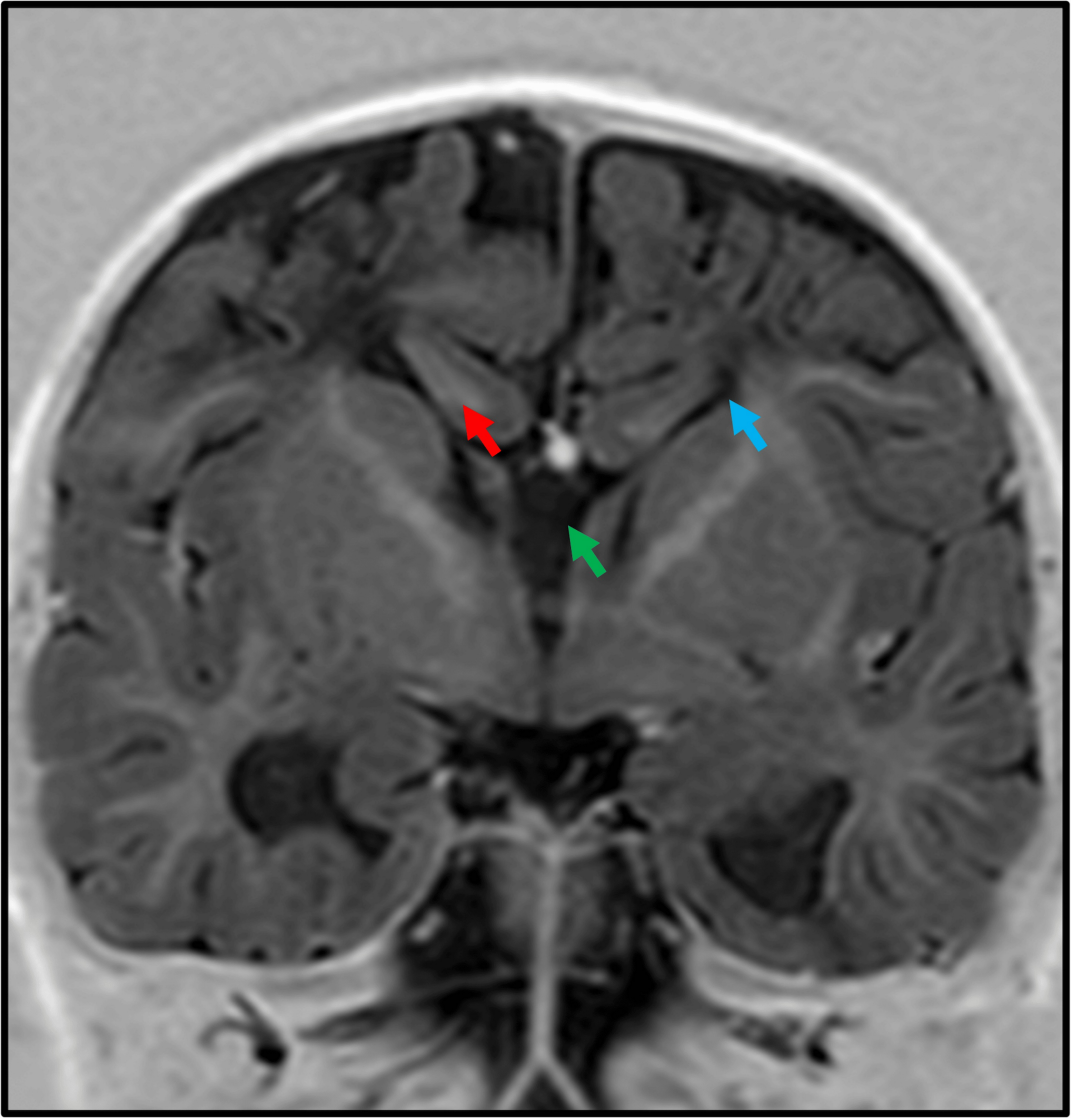
Corpus callosum agenesis with Probst bundle. Coronal T1-weighted inversion recovery image shows bundles of Probst (red arrow) with high riding third ventricle (green arrow). Viking helmet appearance (blue arrow).

The Probst bundle, a common structural anomaly associated with callosal dysgenesis, was identified in 88.9% (eight out of nine) of the complete agenesis cases, compared to only 37.5% (three out of eight) in the partial agenesis group (Figure 5). This association was statistically significant (p < 0.05), indicating a strong correlation between complete agenesis and Probst bundle formation.

Cortical malformation

**Figure 6:**
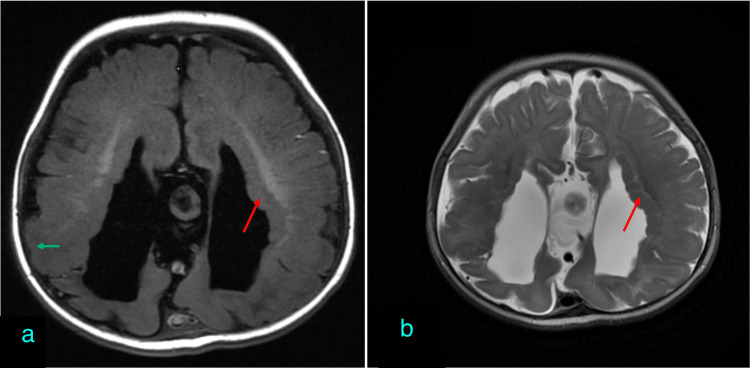
Corpus callosum agenesis with cortical malformation. (a) Axial T1-weighted imaging showing the absence of corpus callosum with diffuse periventricular gray matter heterotopia (red arrow). Gyral abnormality in the form of focal pachygyria in the right parietal lobe (green arrow). (b) Axial T2-weighted imaging showing the absence of corpus callosum with diffuse periventricular gray matter heterotopia (red arrow).

Cortical malformations were observed more frequently in patients with complete agenesis, with 44.4% (four out of nine patients) showing anomalies such as heterotopia and pachygyria (Figure 6). In comparison, only 12.5% (one out of eight patients) with partial agenesis exhibited cortical malformations. This difference was statistically significant (p < 0.05). Heterotopia was the most prevalent cortical malformation associated with complete agenesis in this study.

Other associated anomalies

**Figure 7:**
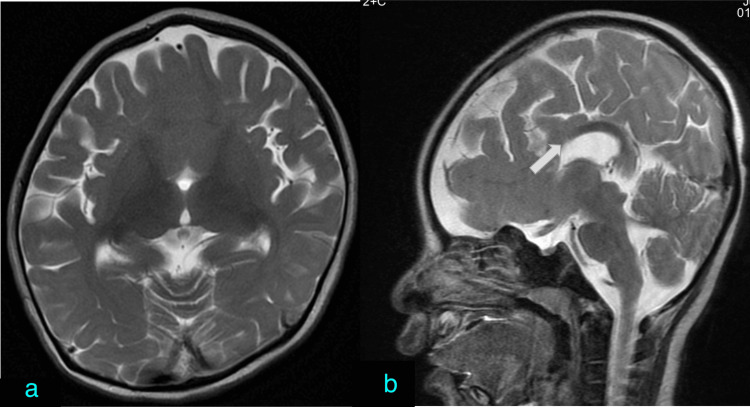
Corpus callosum agenesis with holoprosencephaly. (a) Axial T2-weighted imaging showing fusion of the anterior bilateral frontal lobes with the absence of anterior interhemispheric fissure. (b) Sagittal T2-weighted imaging showing the absence of anterior corpus callosum.

**Figure 8:**
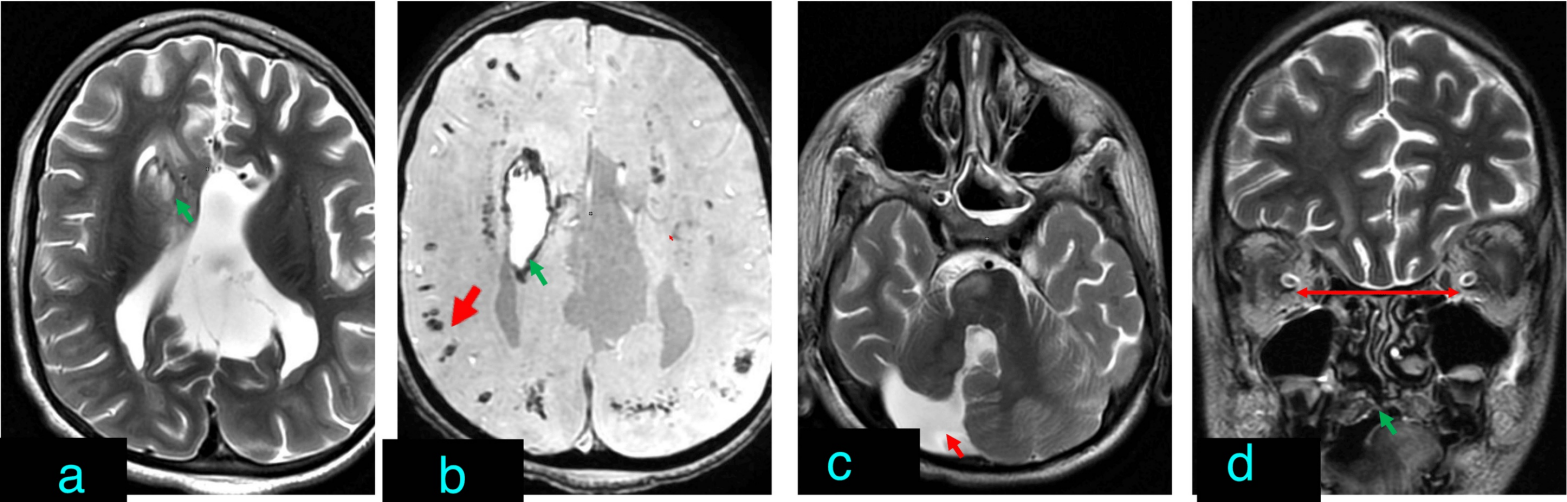
Syndromic association of corpus callosum agenesis with fronto nasal dysplasia, hypertelorism, retrocerebellar cyst and distorted brain stem (a) Axial T2-weighted imaging (T2WI) showing complete agenesis of the corpus callosum and signal alteration in the right-sided deep gray matter (green arrow). (b) Axial T2* (gradient echo) image shows areas of hemorrhage in the subcortical white matter (red arrow) as well as in the deep white and gray matter (green arrow). (c) Axial T2WI showing posterior fossa T2 hyperintense retrocerebellar cyst connecting with the 4th ventricle (red arrow). (d) Coronal T2WI showing cleft palate (green arrow) and hypertelorism (red line).

Additional abnormalities associated with corpus callosum agenesis included holoprosencephaly (n = 1) (Figure 7), Dandy-Walker spectrum malformation (n = 1), optic coloboma (n = 1), HIE changes (n = 2), and a case presenting with a syndromic appearance characterized by frontonasal dysplasia, hypertelorism, retrocerebellar cyst, and brainstem distortion (Figure 8).

## Discussion

Cases of partial and complete corpus callosum agenesis were observed in our study with a ratio of complete agenesis to partial agenesis being 1.12:1. Similar observation was made in a study by Schell-Apacik et al., where complete and partial agenesis of the corpus callosum was found in ratio of 1.25:1 [6]. Szabo et al., in a study on the Hungarian population, observed an equal number of cases of partial and complete agenesis nearly similar to our observation [7].

It is well established in the literature that corpus callosum agenesis is frequently associated with other abnormalities of CNS [8]. These co-existing malformations are responsible for increased morbidity and mortality in patients with corpus callosum agenesis [9]. Patients with agenesis of the corpus callosum without any associated malformation may have normal neuropsychological functioning [10]. As our study was conducted not on the general population but on patients undergoing MRI in tertiary care hospitals, the prevalence of associated anomalies was high, with 76% of patients with corpus callosum agenesis having other brain abnormalities on imaging. In this study, the following associations were seen with corpus callosum agenesis/hypoplasia.

Interhemispheric cysts and lipoma

Embryologically, interhemispheric cysts and lipoma start forming around five weeks of gestation. These may then interfere with the normal formation of the commissural fibers, as commissural fibers are formed later in gestation. Hence, hemispheric cysts and lipomas, which may be the cause behind corpus callosum malformation, are seen as associated with it [11]. The interhemispheric cysts have been classified into two types: type 1 cysts communicate with the ventricles whereas type II cysts show no communication with the ventricle [12]. In our study, two patients with complete corpus callosum agenesis, accounting for 22% of the total cases, had interhemispheric cysts and one patient had lipoma. Our findings are consistent with a previous study conducted by Byrd et al., in which it was observed that 33% of the patients with callosal agenesis had interhemispheric cysts [13]. In a similar study by Hetts et al., it was observed that 14% of patients had interhemispheric cysts and 3% had lipomas associated with corpus callosum agenesis [3].

Commissural abnormalities

Abnormalities of the anterior and hippocampal commissure are commonly associated with corpus callosum agenesis owing to close embryological and developmental association [2]. A total of 44% of patients with complete corpus callosum agenesis had commissural involvement whereas 12.5% of patients with corpus callosum agenesis had commissural involvement. The prevalence of commissural involvement was less in our study as compared to the study by Hetts et al., where it was observed that 83% of patients with complete corpus callosum agenesis (CCA) and 77% of patients with hypoplasia of the corpus callosum (HCC) had abnormalities of the anterior and hippocampal commissure [3]. However, as seen in our study, commissural agenesis is more commonly associated with complete corpus callosum than hypoplastic corpus callosum.

Probst bundles

The fibers that were meant to cross through the corpus callosum into the other hemisphere instead run in a parallel configuration in their own hemisphere. These fiber bundles are called Probst bundles. Owing to this fact, Probst bundles are the most common structural anomalies associated with callosal dysgenesis. Also, they are more common in complete agenesis compared to partial agenesis as partial agenesis allows passage of white matter tracts across the hemisphere through the formed corpus callosum [14]. In our study, 89% of patients with complete agenesis of the corpus callosum had Probst bundles compared to 37.5% of patients with hypoplastic corpus callosum. This is in accordance with a previous study by Hetts et al., in which 88% of patients with absent corpus callosum had Probst bundles whereas 38% with partial agenesis of the corpus callosum had Probst bundles [3].

Malformation in cortical development

Various cortical malformations are associated with corpus callosum agenesis like lissencephaly, polymicrogyria, and schizencephaly. In a study by Schell-Apacik et al., polymicrogyria was observed in 29% of patients with complete agenesis of the corpus callosum and in 7.6% of patients with partial agenesis of the corpus callosum [6]. Pachygyria was seen in 25% of patients with complete agenesis of the corpus callosum and in 7.6% of patients with partial agenesis of the corpus callosum whereas heterotopia was seen in 14% of patients with complete agenesis of the corpus callosum and in 1% of patients with partial agenesis of the corpus callosum [5]. Another study by Bedeschi et al. demonstrated that 33% of patients with complete agenesis of the corpus callosum had a malformation of cortical development [15]. In our study, the association with cortical malformation was observed to be more than in the previous studies, with cortical malformations in 44% of the cases with complete agenesis and 12.5% of cases with partial agenesis. This increased observation of cortical malformation could be attributed to the fact that patients with more severe abnormalities are more likely to present to a tertiary care institute in a resource-limited region. The most commonly associated cortical malformation in our study was heterotopia, followed by pachygyria.

Ventricular distortion

In cases of absence of corpus callosum, the lateral ventricle appears to have a parallel configuration and the third ventricle appears high riding. These variations are simply morphological changes due to the absence of the corpus callosum and are not malformations on their own [16]. However, abnormalities of the ventricle like colpocephaly are frequently associated with corpus callosum agenesis [17].

Abnormal ventricles are more common in the agenesis of the corpus callosum than partial agenesis of the corpus callosum. In our study, abnormal ventricles were seen in 88.9% of cases of complete agenesis and 40% of cases of partial agenesis. The percentage of patients with ventricular abnormality was less in our study as compared to the study by Hetts et al., in which abnormal cerebral ventricles were seen in 96% of cases with agenesis of the corpus callosum and 83% of cases with partial agenesis of the corpus callosum. The association of ventricular abnormality was seen more with complete agenesis than with partial agenesis group, similar to the findings in previous studies [3].

Other associated abnormalities

Dandy-Walker spectrum malformation has an association with corpus callosum agenesis. In our study, one case of complete corpus callosum agenesis had Dandy-Walker malformation whereas another case of complete corpus callosum agenesis had a retro-cerebellar cyst. The association between corpus callosum agenesis and posterior fossa malformation has been described in previous studies as well. Various causes have been attributed to this association, including earlier insults to the developing rhombencephalon [17].

In the case of alobar holoprosencephaly, as both the cerebral hemispheres are fused, the scope of fibers connecting the two hemispheres does not exist and hence corpus callosum is absent. In semi-lobar holoprosencephaly, the frontal lobes are fused, hence anterior corpus callosum is absent. In lobar holoprosencephaly, there is a fusion of antero-basal frontal lobes and an absence of adjacent corpus callosum [18]. It is to be noted that in partial agenesis of the corpus callosum, the later-formed posterior segments of the corpus callosum are absent whereas in the holoprosencephaly spectrum, the anterior corpus callosum is absent. As in holoprosencephaly, the anterior frontal horns are fused and the absence of corpus callosum is seen in the fused region [19]. The same was observed in our case with lobar holoprosencephaly where anterior frontal lobes were fused with the absence of the anterior part of the corpus callosum.

Various genetic syndromes are associated with corpus callosum agenesis [20]. In our study, a syndromic appearing case with frontonasal dysplasia, retro-cerebellar cyst, hypertelorism, and brainstem distortion was observed. Based on clinical features, the possibility of ciliopathy syndrome was kept. However, a detailed genetic evaluation of the case was beyond the scope of this study.

Limitations

This study has a few limitations that should be acknowledged. First, the sample size was relatively small, which may limit the generalizability of our findings. A larger sample size would be needed to validate these results and provide more robust statistical power for detecting associations between corpus callosum agenesis and other brain malformations. Second, the study did not include a genetic evaluation to investigate potential genetic causes underlying the malformations and associated syndromes. Future studies should consider incorporating genetic testing to provide a more comprehensive understanding of the etiology and potential hereditary factors involved in these complex cases.

## Conclusions

This study provides insights into the MRI evaluation of corpus callosum malformations and imaging of associated anomalies. The findings reveal a significant correlation between the type of agenesis (complete or partial) and the prevalence of associated brain abnormalities. Complete agenesis of the corpus callosum is more commonly associated with commissural abnormalities, ventricular distortions, cortical malformations, midline cysts, and Probst bundle formation compared to partial agenesis. The study comprehensively shows all the common associations with corpus callosum agenesis and their relative prevalence in a tertiary care center in northern India.

## References

[1] Atlas SW, Zimmerman RA, Bilaniuk LT, Rorke L, Hackney DB, Goldberg HI, Grossman RI (1986). Corpus callosum and limbic system: neuroanatomic MR evaluation of developmental anomalies. Radiology.

[2] Paul LK (2011). Developmental malformation of the corpus callosum: a review of typical callosal development and examples of developmental disorders with callosal involvement. J Neurodev Disord.

[3] Hetts SW, Sherr EH, Chao S, Gobuty S, Barkovich AJ (2006). Anomalies of the corpus callosum: an MR analysis of the phenotypic spectrum of associated malformations. AJR Am J Roentgenol.

[4] Das JM, Geetha R (2023). Corpus callosum agenesis. StatPearls.

[5] Jeret JS, Serur D, Wisniewski KE, Lubin RA (1987). Clinicopathological findings associated with agenesis of the corpus callosum. Brain Dev.

[6] Schell-Apacik CC, Wagner K, Bihler M (2008). Agenesis and dysgenesis of the corpus callosum: clinical, genetic and neuroimaging findings in a series of 41 patients. Am J Med Genet A.

[7] Szabó N, Gergev G, Kóbor J, Bereg E, Túri S, Sztriha L (2011). Corpus callosum anomalies: birth prevalence and clinical spectrum in Hungary. Pediatr Neurol.

[8] Stoll C, Dott B, Roth MP (2019). Associated anomalies in cases with agenesis of the corpus callosum. Am J Med Genet A.

[9] Palmer EE, Mowat D (2014). Agenesis of the corpus callosum: a clinical approach to diagnosis. Am J Med Genet C Semin Med Genet.

[10] Siffredi V, Anderson V, McIlroy A, Wood AG, Leventer RJ, Spencer-Smith MM (2018). A neuropsychological profile for agenesis of the corpus callosum? Cognitive, academic, executive, social, and behavioral functioning in school-age children. J Int Neuropsychol Soc.

[11] Sarnat HB (2008). Embryology and malformations of the forebrain commissures. Handb Clin Neurol.

[12] Barkovich AJ, Simon EM, Walsh CA (2001). Callosal agenesis with cyst: a better understanding and new classification. Neurology.

[13] Byrd SE, Radkowski MA, Flannery A, McLone DG (1990). The clinical and radiological evaluation of absence of the corpus callosum. Eur J Radiol.

[14] Tang PH, Bartha AI, Norton ME, Barkovich AJ, Sherr EH, Glenn OA (2009). Agenesis of the corpus callosum: an MR imaging analysis of associated abnormalities in the fetus. AJNR Am J Neuroradiol.

[15] Bedeschi MF, Bonaglia MC, Grasso R (2006). Agenesis of the corpus callosum: clinical and genetic study in 63 young patients. Pediatr Neurol.

[16] Barkovich MJ (2022). Pediatric brain maturation and migration disorders. Diagnostics (Basel).

[17] Barkovich AJ, Norman D (1988). Anomalies of the corpus callosum: correlation with further anomalies of the brain. AJR Am J Roentgenol.

[18] Ramakrishnan S, Das JM (2017). Holoprosencephaly. Obstetric Imaging: Fetal Diagnosis and Care, 2nd Edition.

[19] Raam MS, Solomon BD, Muenke M (2011). Holoprosencephaly: a guide to diagnosis and clinical management. Indian Pediatr.

[20] Hofman J, Hutny M, Sztuba K, Paprocka J (2020). Corpus callosum agenesis: an insight into the etiology and spectrum of symptoms. Brain Sci.

